# VTCdb: a gene co-expression database for the crop species *Vitis vinifera* (grapevine)

**DOI:** 10.1186/1471-2164-14-882

**Published:** 2013-12-16

**Authors:** Darren CJ Wong, Crystal Sweetman, Damian P Drew, Christopher M Ford

**Affiliations:** 1 School of Agriculture, Food and Wine, University of Adelaide, Adelaide 5064, South Australia, Australia; 2 Department of Plant and Environmental Sciences, Section for Plant Biochemistry, University of Copenhagen, Frederiksberg 1871, Denmark

## Abstract

**Background:**

Gene expression datasets in model plants such as *Arabidopsis* have contributed to our understanding of gene function and how a single underlying biological process can be governed by a diverse network of genes. The accumulation of publicly available microarray data encompassing a wide range of biological and environmental conditions has enabled the development of additional capabilities including gene co-expression analysis (GCA). GCA is based on the understanding that genes encoding proteins involved in similar and/or related biological processes may exhibit comparable expression patterns over a range of experimental conditions, developmental stages and tissues. We present an open access database for the investigation of gene co-expression networks within the cultivated grapevine, *Vitis vinifera*.

**Description:**

The new gene co-expression database, VTCdb (http://vtcdb.adelaide.edu.au/Home.aspx), offers an online platform for transcriptional regulatory inference in the cultivated grapevine. Using condition-independent and condition-dependent approaches, grapevine co-expression networks were constructed using the latest publicly available microarray datasets from diverse experimental series, utilising the Affymetrix *Vitis vinifera* GeneChip (16 K) and the NimbleGen Grape Whole-genome microarray chip (29 K), thus making it possible to profile approximately 29,000 genes (95% of the predicted grapevine transcriptome). Applications available with the online platform include the use of gene names, probesets, modules or biological processes to query the co-expression networks, with the option to choose between Affymetrix or Nimblegen datasets and between multiple co-expression measures. Alternatively, the user can browse existing network modules using interactive network visualisation and analysis via CytoscapeWeb. To demonstrate the utility of the database, we present examples from three fundamental biological processes (berry development, photosynthesis and flavonoid biosynthesis) whereby the recovered sub-networks reconfirm established plant gene functions and also identify novel associations.

**Conclusions:**

Together, we present valuable insights into grapevine transcriptional regulation by developing network models applicable to researchers in their prioritisation of gene candidates, for on-going study of biological processes related to grapevine development, metabolism and stress responses.

## Background

The cultivated grapevine *Vitis vinifera* is one of the most highly valued horticultural crops in the world, and amongst the earliest domesticated fruit crops in human history. The global production of grapes in 2011 was 70 million tonnes, harvested over approximately 7 million hectares of land, making the grapevine the most widely cultivated fruit species [1]. Quality attributes of grapes, including aroma, flavour, colour and texture characteristics, have a profound impact on the fruit and wine and therefore on the value of the crop itself. An in-depth understanding of gene expression and the regulation of metabolic pathways controlling various aspects of grapevine development and berry metabolism could provide insights into the genetic factors influencing fruit quality and ultimately inform future vineyard germplasm and cultural practices.

Functional genomics studies in plants have contributed to a systems-level understanding of how genes function and how an underlying biological process is governed by the cooperation of a set of genes. Genome sequencing of two grapevine cultivars [2,3] and successive improvements on genome assembly and prediction [4-6] have been invaluable for gene discovery, while application of high throughput technologies such as microarrays has enabled large-scale transcriptional analysis in grapevine. During the pre-genomic period, sequences selected from Genbank, expressed sequence tags and the NCBI RefSeq grapevine transcripts were the main sources for the design and annotation of the grapevine 16 K Affymetrix Genechip (http://www.affymetrix.com), with approximately one third of the transcriptome represented on the array based on the 12X v1 gene annotation [6]. The grapevine 29 K Nimblegen whole-genome array, (http://www.nimblegen.com), which represents approximately 29,000 genes (>95% of the predicted transcriptome) is the most well-developed of the grapevine microarray platforms. To date, microarray studies feature numerous experiments, including different stages of berry development for various cultivars [7,8], a range of grapevine tissues [9,10] and the application of various biotic and abiotic stressors [11,12]. A survey from gene expression data repositories including the Gene Expression Omnibus [13] and Arrayexpress [14] revealed that a large number of expression datasets have been generated from plants, especially *Arabidopsis thaliana*, *Glycine max* (soybean) and *Oryza sativa* (rice), and these also involve diverse experimental conditions. Although these gene expression datasets have been primarily generated within a particular experimental context, the accumulation of large numbers of expression profiles has offered additional capabilities. These include comparative genomics between plant species, screening and functional assignment of gene candidates, the discovery of novel DNA motifs, and the dissection of regulatory networks. One technique that has proved invaluable in this role is that of gene co-expression analysis (GCA).

GCA is based on the notion that genes involved in similar or related processes may exhibit similar expression patterns over a range of experimental conditions [15,16]. This “guilt by association” principle has been initially applied to gain insights into co-expressed gene modules within an organism [17,18], to assign novel gene functions previously not ascribed to any biological processes, and to understand the evolution of gene expression and diversity across species and kingdoms [19,20]. A ‘condition-independent’ GCA derived from a large dataset compiled from various experimental conditions has been adopted in many studies for convenience while providing a global overview of gene-to-gene relationships [21-23]. However, drawbacks to the condition-independent approach include the complexity of drawing biological insights and the potential loss of co-expression relationships due to variation between the numerous experimental conditions. Alternatively, the ‘condition-dependent’ approach, which draws upon GCA derived from smaller and predefined datasets (conditions), provides an additional opportunity to test specific hypotheses or to gain biological insights in an underlying condition [24,25]. However, when too few sample datasets are chosen, noise inherent in the microarray data will also affect the results obtained from ‘condition-dependent’ GCA. Nevertheless, both ‘condition-independent’ and ‘condition-dependent’ approaches have proven useful in many co-expression studies in plants [16].

Within a co-expression network, genes and similarity relationships (commonly represented by correlation coefficients) can be visualised as “nodes” and “edges” respectively. The connection of two nodes by an edge indicates a similar expression profile of the nodes according to a particular similarity metric. For a given set of genes, the collection of these nodes and edges forms a network. Visualisation of the co-expression network enables the identification and description of densely connected gene clusters, referred to as modules, and an assessment of biological relevance can be achieved by investigating the functions of genes within each module [15,26]. Many graph clustering algorithms have been developed with the aim of extracting functional modules comprising densely connected groups of nodes (representing co-expressed genes). Such algorithms can be classified as density-based and local search algorithms, hierarchical clustering, and other optimization-based algorithms [27]. In addition to the model plant *Arabidopsis,* these algorithms have also been applied to study co-expression networks in important crop species such as rice, barley and soybean [21,28], with databases developed to store inferred modules and provide a user-friendly resource for plant biologists. Examples of outcomes reported using the “guilt by association” principle include the identification of genes involved in cellulose biosynthesis [29] and transcription factors (TFs) involved in glucosinolate regulation in *Arabidopsis*[30].

In the present study, over 800 publicly available microarray datasets related to the *V. vinifera* L. transcriptome were selected to construct global co-expression networks (GCNs), consisting of 463 datasets from the Nimblegen whole-genome arrays and 403 datasets from the 16 K Affymetrix Genechip arrays. A combination of correlation rank transformation and graph-clustering approaches was used. With particular emphasis on the GCN constructed using the Nimblegen whole-genome array, we demonstrate the utility of this *V. vinifera* GCN using selected examples where we confirm well-characterised biochemical pathways, and infer potential novel gene functions and processes. A dedicated grapevine gene co-expression database, named VTCdb (http://vtcdb.adelaide.edu.au/Home.aspx), equipped with functional enrichment and visualisation capabilities, has been made available to the public to query and browse the associated GCN.

## Construction and content

### Data acquisition and processing

Publicly available grapevine 29 K NimbleGen whole-genome (http://www.nimblegen.com) and 16 K Affymetrix Genechip (http://www.affymetrix.com) microarray datasets were retrieved from Gene Expression Omnibus [13], Arrayexpress [14] and PLEXdb [31]. Summaries of the experiments and associated metadata are given in Additional files 1 and 2, detailing the 481 (Nimblegen) and 451 (Affymetrix) arrays (containing approximately 29,000 and 16,000 probesets respectively) that were used for subsequent analysis. Raw intensity data from both platforms were separately background-adjusted, quantile-normalised and summarised using the RMA method in R (http://www.r-project.org) using the ‘oligo’ package [32]. Potential outlier arrays were removed by visual inspection of raw perfect match data and iteratively discarding arrays that failed the quality control test (where expression values deviated significantly from the relative log expression and the normalised unscaled standard error) leaving 463 (Nimblegen) and 403 (Affymetrix) arrays for subsequent analysis (see Additional file 2). A survey of the underlying experimental conditions represented by the arrays can be assembled into a general category (‘All’ datasets) covering a broad range of treatments and plant development stages such as tissue development, stress and vineyard management strategies. Additionally, two condition-specific datasets were established for the 29 K Nimblegen datasets, one for berry-related tissues and treatments only, and one for stress-related processes (biotic and abiotic) across the whole vine (Additional file 2). The number of arrays corresponding to arrays for ‘All’ , ‘Berry’ and ‘Stress’ datasets are 463, 305 and 59, respectively. Together, this provided a broad basis for inferring both condition- independent and dependent gene co-expression relationships in grapevine. Separate GCNs were generated for all, berry- and stress-related datasets by applying the procedure below.

### Gene co-expression network construction

Correlations between all mapped probesets were calculated using Pearson’s correlation coefficient (PCC) and Spearman’s correlation coefficient (SCC) as measures of similarity between expression profiles. Additionally, the mutual co-expression relationships between all gene pairs were calculated (without applying any cut-offs) by first transforming raw correlation values (PCC and SCC) into highest reciprocal ranks (HRR) [33] and mutual ranks (MR) [34]. Rank-based networks are robust and offer advantages over correlation-based networks [34,35]. Such approaches have been frequently applied to retain weak but significant co-expression relationships and circumvent the unequal distribution of gene correlations for some genes when applying a fixed similarity threshold. This index of co-expression (HRR and MR) serves as a basis for ranking co-expressed genes when using a ‘guide gene’ approach to query the network. In this study, we focussed our attention on the mutual co-expression relationships derived from PCC values for simplicity, and because the Gene ontology (GO) prediction performance of transformed ranks from PCC and SCC values were similar [34]. An estimation of the statistical significance of mutual co-expression ranks [21] showed that HRR and MR values ≤ 350 and 200 respectively were significant (*P* < 0.01), and therefore these were applied as a generalised threshold for obtaining biological relevant relationships in grapevine.

### Graph clustering and meta-network construction

To identify modules of densely connected nodes, the Heuristic Cluster Chiselling Algorithm (HCCA) [33] and Markov clustering (MCL) [36] techniques were applied. With an input network of HRR ≤ 30, we first assigned weights of 0.2, 0.067, and 0.04 to HRR scores of 10, 20 and 30, respectively. Parameters were adjusted to a desired step size of 3 and cluster size between 40 and 400 for HCCA and an inflation value of 1.2 for MCL using Python 2.7.3 (http://www.python.org) and MCL version 12–068 (http://micans.org/mcl/), respectively. To depict the relationships between modules generated, we first calculated the total edge weights shared between any two connected modules and assigned an empirically derived statistical significance (*P*-value) by permutation test according to [21]. The various grapevine meta-networks were constructed with edges connecting modules at a significance of *P* < 0.01.

### Functional enrichment and expression specificity analysis

To assist with the categorisation of co-expressed genes and partitioned modules according to their potential function or processes, we assessed the modules for enrichment primarily for GO terms in R (http://www.r-project.org) using the ‘gProfileR’ package [37] to interface g:profiler (http://gprofiler.at.mt.ut.ee/gprofiler/). Enrichment for GO terms was validated using the hypergeometric distribution adjusted by set count sizes (SCS) for multiple hypothesis correction. SCS threshold considers the hierarchal structure of GO in an underlying organism and prioritises truly significant results (while removing enriched false positive GO terms) [37]. GO terms were considered significant if the adjusted *P*-values (SCS) < 0.05 and there were at least two genes associated with the same annotation. Network representation of GO terms was prepared using GO-module webserver [38]. Expression specificities of individual probesets and modules were determined using the Std2Gx procedure [28]. Expression specificity index values > 1 and > 5 indicates the gene is well and specifically expressed in the corresponding experimental condition respectively, as compared with other genes and array samples. Expression specificity of modules is expressed as the percentage of module members specifically expressed in a particular tissue/condition (and across all arrays) with an expression specificity index above 1.

### Gene annotations and network visualization

The latest grapevine gene and probeset annotations based the 12X v1 prediction were obtained from Vitisnet [4,39] and mappings for probesets containing functional annotation and categorization of predicted genes (chromosome location, predicted function, subcellular localization, orthology and pathway level information) were used. CytoscapeWeb [40] was used to visualise nodes and edges and their attributes.

## Utility

### VTCdb web interface and content

**Figure 1:**
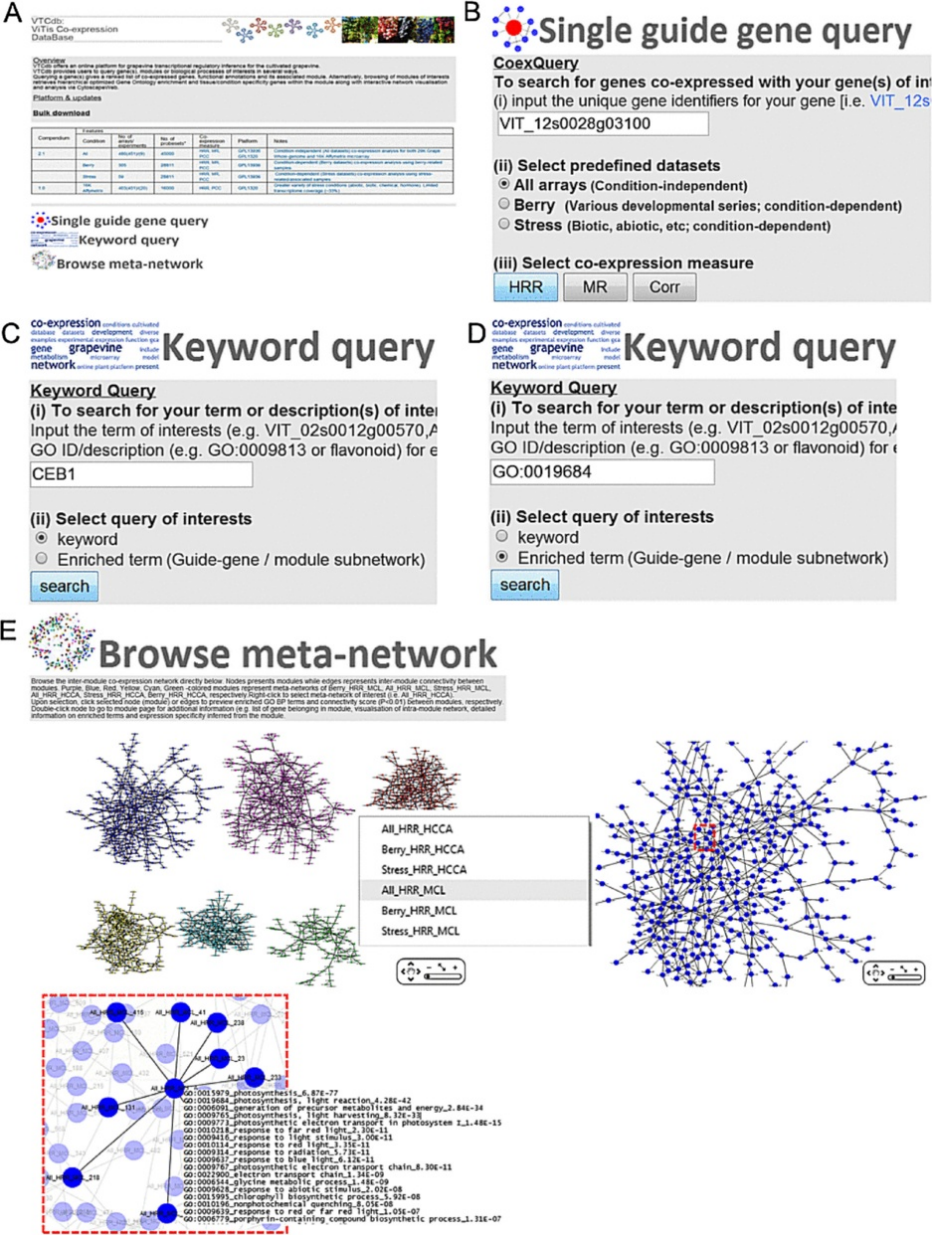
**Screenshots of VTCdb home page displaying different search forms.** The home page **(A)** contains a brief introduction into the VTCdb webserver. To begin queries, the user must select ‘Single guide gene query’, which will cascade **(B)** and ‘Keyword query’ which will cascade **(C)** and **(D)**. Selecting ‘Browse meta-network’ will cascade the meta-network interface **(E)**.

VTCdb (http://vtcdb.adelaide.edu.au/Home.aspx) can be accessed via a user-friendly web interface that includes tools to query, browse and visualise the co-expression network genes and modules. VTCdb runs on an Internet Information Server (IIS, version 7.0) containing data tables stored in MS Access (Microsoft Corporation Inc., USA). The web pages were built using a combination of ASP.NET 4.0, Javascript and Jquery 1.4. The VTCdb home page contains several search forms to retrieve co-expressed genes and related information, including a co-expressed genes search, a keyword search, an enriched term search, and an option to browse the meta-network interfaces (Figure 1A-E). With the current release, queries can be performed against the grape co-expression network inferred using the Nimblegen grape genome arrays (default) or the grape Affymetrix Genechip arrays.

Under the single guide gene query, when a gene identifier (i.e. VIT_ code) is used as a query using the ‘CoexQuery’ field, the user can select the various predefined conditions (‘All’ , ‘Berry’ and ‘Stress’) followed by the preferred co-expression measure (‘HRR’ , ‘MR’ and ‘CORR’) (Figure 1B). Users will be re-directed to the co-expressed genes result page, ‘CoexQuery result’ for the chosen gene, with the chosen dataset and co-expression measure.

**Figure 2:**
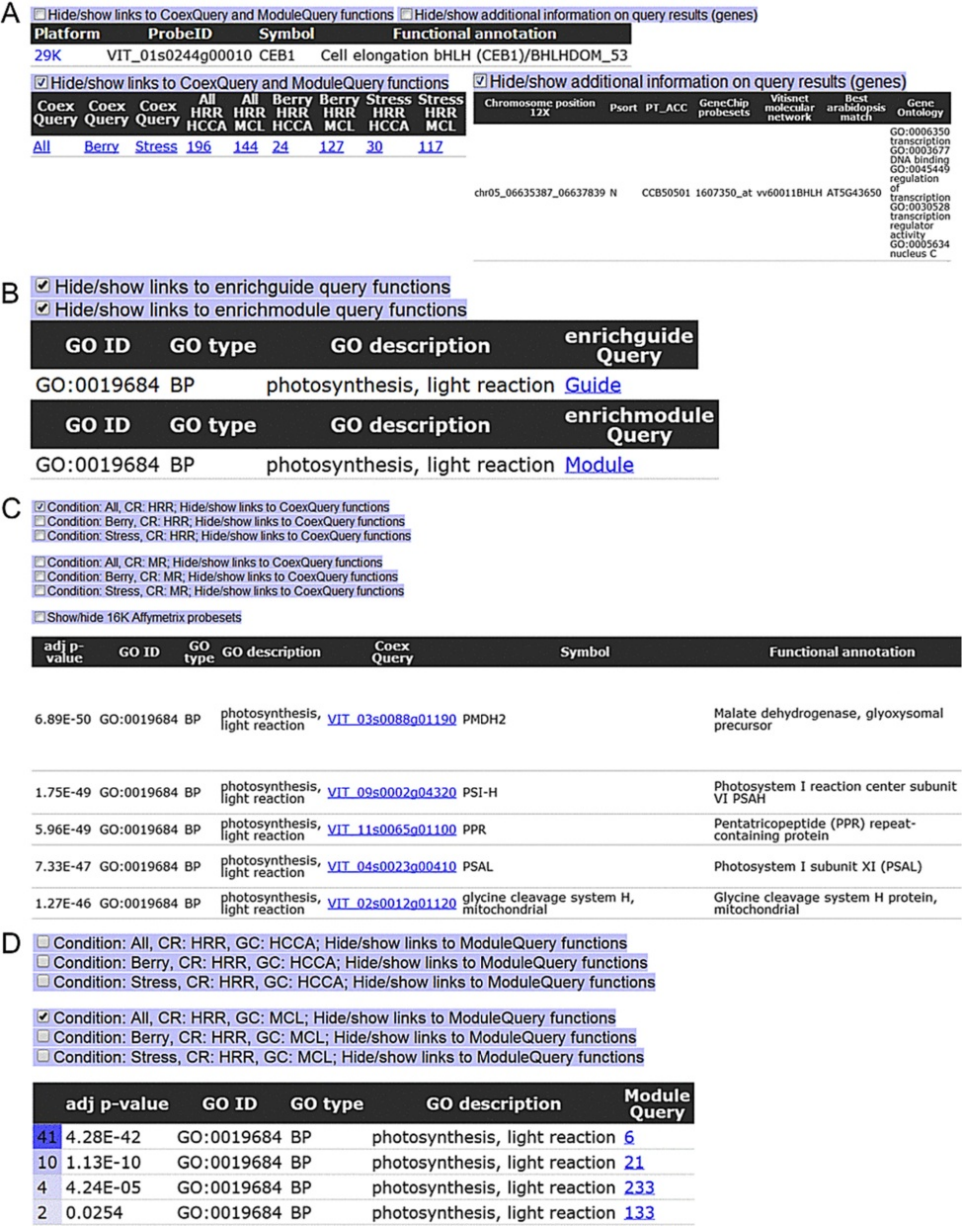
**Screenshots of VTCdb pages relevant to the utility and output of keyword query, enrich guide and enrich module searches.** The ‘Keyword’ results pages **(A)**, includes probesets and annotations with matching terms, as well as links to the ‘Co-expressed genes’ and ‘Module’ result page. The ‘Enrich keyword query’ result page **(B)** reports matching GO query terms (ID/ description) and provides links to the ‘Enrich guide’ **(C)** and ‘Enrich module’ **(D)** results pages. The ‘Enrich guide’ result page **(C)** reports genes in which their co-expressed genes are functionally enriched with GO query terms of interests. The page contains checkboxes to select criteria of interest and a table contains functional annotations of genes, enrichment values of GO query terms and gene links to perform ‘Single guide gene query’ with the selected parameters of interest. Similarly, the ‘Enrich module’ results page **(D)** reports modules in which genes within the module are functionally enriched with GO queries of interest. The page contains checkboxes to select criteria of interest and a table containing enrichment values of GO query terms and module links to perform ‘Module query’ with the selected parameters of interest.

To search for a term or description associated with a gene, users should input the keyword of interest and select the ‘keyword’ option (Figure 1C). The keyword query tool will perform a broad search across various fields (i.e. gene identifiers, symbols, functional annotations, associated ontology/pathway terms) within the gene annotation tables for matches and redirects users to the ‘keyword results’ page containing associated genes, information and links for further downstream analysis using the various toggles (Figure 2A). To search for enrichment descriptors (GO ID or term; if present) within sub-networks of guide-genes and modules, select the ‘enriched term’ button (Figure 1D). Users will be redirected to the ‘enriched keyword’ page which provides an interface with associated links to the ‘enrich keyword query’ results page (Figure 2B). The ‘enrich guide’ (Figure 2C) and ‘enrich module’ (Figure 2D) results pages contain lists of enrichment values for the given GO term of interest and links to the ‘CoexQuery’ and ‘module results’ pages, respectively. Checkboxes allow users to choose between these various parameters. Additionally, via the CytoscapeWeb interface, users are able visualise meta-networks (i.e. All_HRR_MCL) and browse nodes (modules in this case) within the meta-network (Figure 1E). By moving the cursor over the module of interest, significantly connected modules become highlighted and details of the module size, number of edges and enriched GO BP terms are given. Double clicking the modules takes users to the module result page, with detailed information on the module of interest.

**Figure 3:**
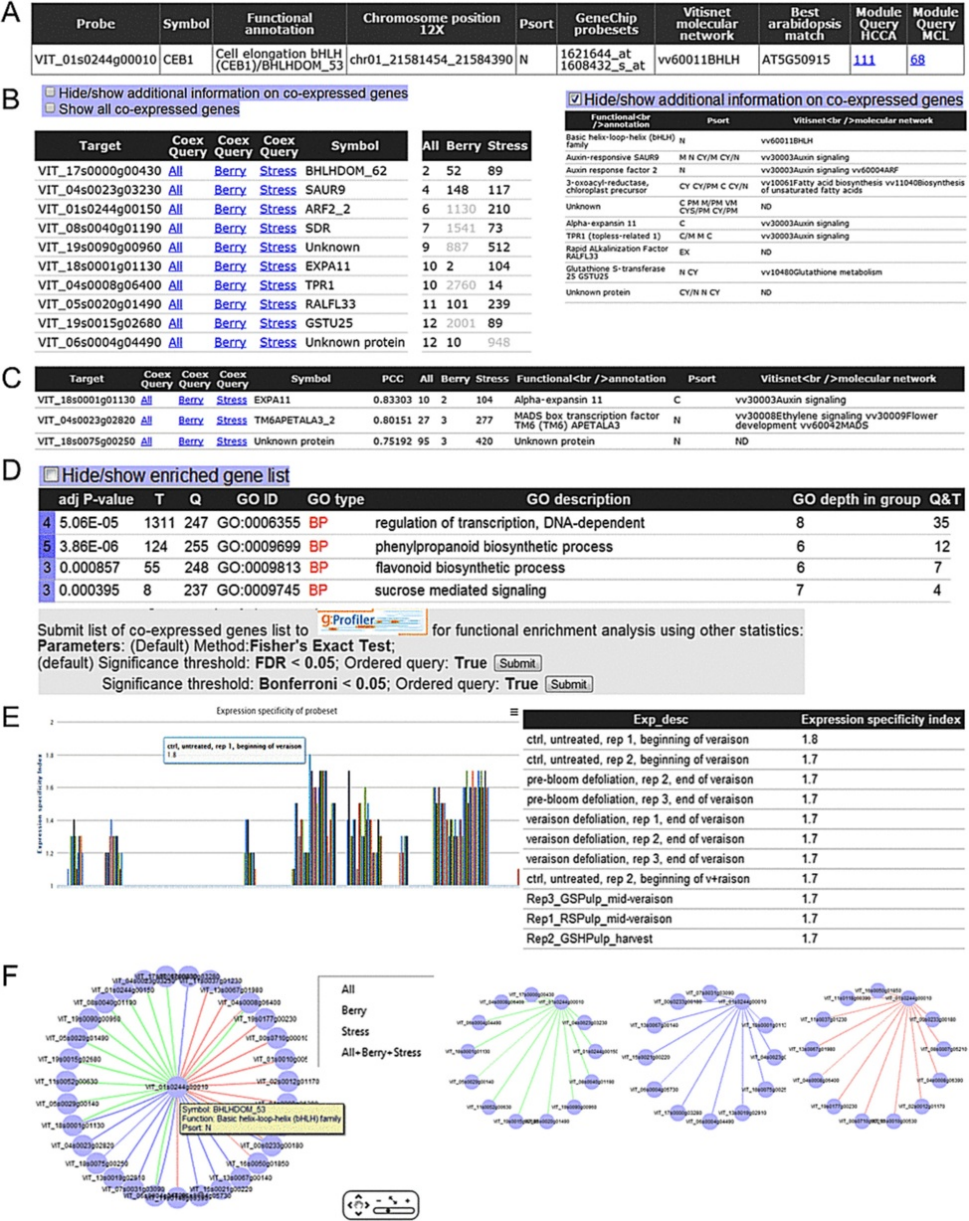
**Screenshots of VTCdb pages relevant to the output of the ‘Co-expressed genes’ result page.** The ‘Co-expressed genes’ result page contains functional annotation of query gene **(A)** and a list of condition-independent and/or -dependent co-expressed genes sorted by ascending metric of interests **(B and C)**, functionally enriched terms (when available) and additional options to send the gene lists for GO enrichment analysis using various parameters **(D)**, expression specificity of query gene in graph and table format **(E)** and interactive visualisation of gene co-expression network **(F)**.

**Figure 4:**
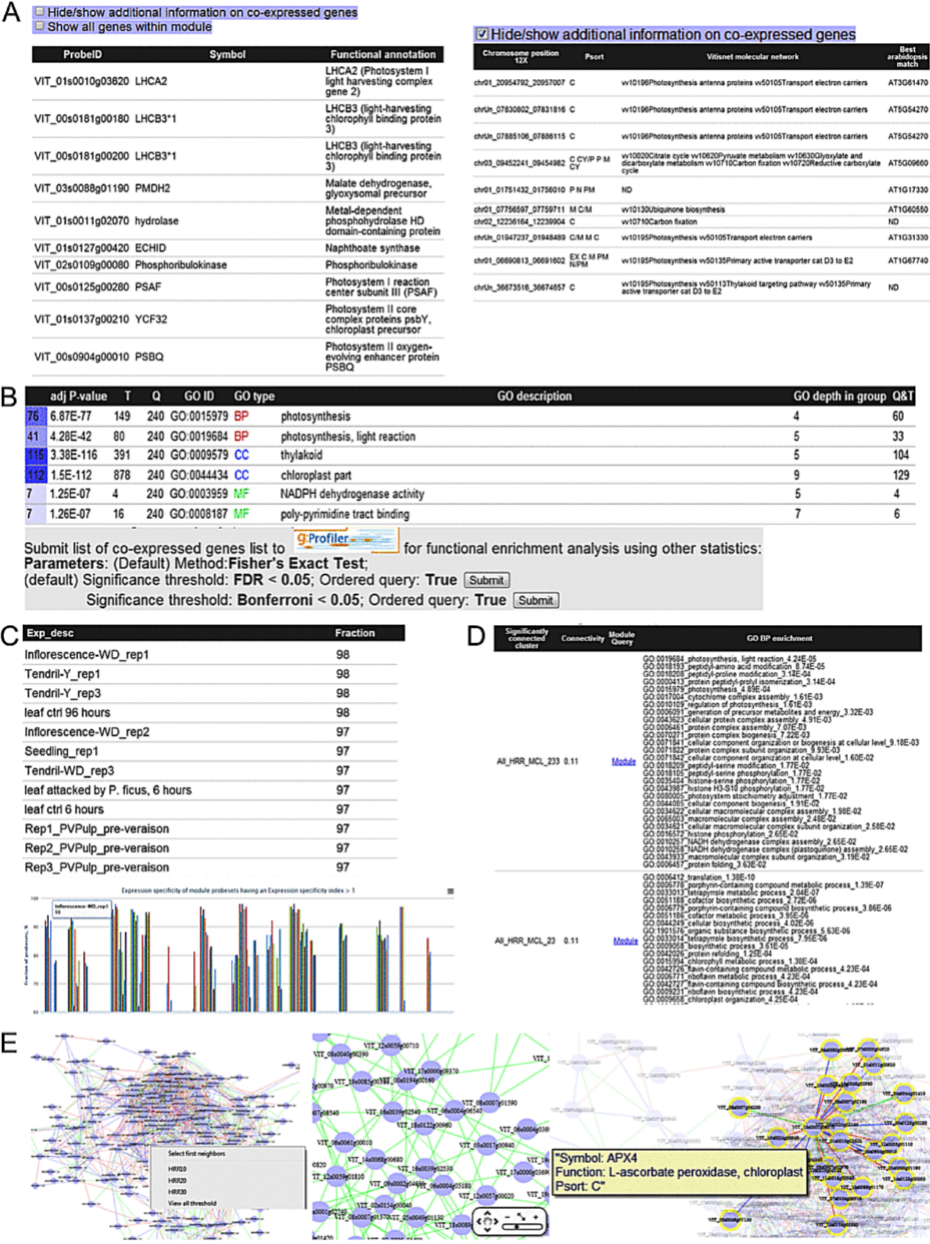
**Screenshots of VTCdb pages relevant to the output of the ‘Module’ result page.** The ‘Module’ results page contains a list of genes belonging to the module **(A)**, functionally enriched terms (when available) and additional options to send the gene lists for GO enrichment analysis using various parameters **(B)**, expression specificity of genes belonging to associated module in graph and table format **(C)**, a list of significantly connected modules with query module **(D)** and interactive visualisation of gene co-expression network **(E)**.

The co-expressed genes ‘CoexQuery’ result page contains a table with detailed information (gene annotation) on query genes and the associated module (when identifiable using HCCA or MCL) to which the query gene belongs (Figure 3A). Next, a list containing information on co-expressed genes (ranked in ascending order of co-expression strength of desired condition) is displayed (Figure 3B). In brief, the table displays the top 50 co-expressed genes, links to ‘CoexQuery’ result pages for each gene, and the co-expression strength in the conditions of interest (i.e. ‘all’ , ‘berry’ and ‘stress’ networks, where applicable). Insignificant ranks (HRR > 750, MR > 550; *P* > 0.05) in corresponding conditions are coloured in grey. Using various checkboxes, the table can be fully expanded to show all significant co-expressed genes and additional annotations (i.e. predicted functional annotation, localization, and molecular network). Clicking on a sub-column in the ‘CoexQuery’ field (i.e. ‘all’ , ‘berry’ or ‘stress’) in any row will then open a new ‘CoexQuery’ result page with the selected network for the corresponding gene. Columns can be sorted by clicking the headers of the table, which provides flexibility for the user interested in ranking the co-expressed genes list according to other conditions of interests (Figure 3C), indexes of co-expression (e.g. PCC) or grouping molecular network annotations. Functional (GO) category over-representation of co-expressed genes will be displayed (if present) in a table sorted in ascending enrichment *P*-values for every GO category along with test statistics for the enriched GO term and information of the enriched genes (Figure 3D). Alternatively, users can submit the list of co-expressed genes to gprofiler for functional enrichment analysis. Expression specificity of the query gene in various experimental conditions is displayed in a graph format (Figure 3E). By moving the cursor over the graphs, users can see the underlying experimental conditions and expression specificities of respective modules. Alternatively, the expression specificity table for the guide gene can be viewed when the hide/show expression specificity is checked. A network representation of the top 10 co-expressed genes with the query (guide) gene from various predefined datasets can be visualised using Cytoscapeweb (Figure 3F). Additional information on annotations and co-expression conditions can also be viewed by placing the cursor over the nodes or edges, respectively. In addition, users can view the top 10 co-expressed genes for a condition-dependent network by right-clicking and selecting the preferred condition. Similarly, the ‘Module result’ page (Figure 4) is composed of four separate tables, the first containing genes belonging to the module (Figure 4A), the second containing GO enriched terms (sorted by type and SCS significance) (Figure 4B), the third containing expression specificity of the inferred module (Figure 4C), and the final table is a list of significantly connected modules (Figure 4D). Again, various checkboxes can be used to hide/expand additional information pertaining to genes, modules and enrichment terms. The expression specificity graph allows the user to visualise tissue/sample conditions under which the majority of the probesets within that module are specifically expressed (Figure 4C). Additionally, network visualisation tools for networks analysis are provided using the CytoscapeWeb application (Figure 4E). Functions such as displaying node/edge annotations, highlighting first-neighbours of nodes, and visualisation at different cut-off parameters enable manipulation of the co-expression network according to user preference.

## Discussion

To demonstrate the applicability and robustness of the VTCdb web server for co-expression studies, we present some examples for the use of VTCdb query tools in the analysis of well-characterised biological processes and highlight gene co-expression networks that may be of biological interest in future grapevine research.

### Example application I: grapevine berry development

Grapevine fruit development can be broken into 3 phases by chronological sequence: berry formation, veraison and berry ripening, reviewed in [41]. Each of these involves specific changes in gene expression, biochemical, compositional and physiological properties of the berry. For example, processes involving cell wall reorganization are crucial during periods of rapid cell division and elongation (during berry formation) and softening (during berry ripening). Accumulating evidence suggests that the involvement of various activities of grapevine expansins (among others) are crucial in regulating cell wall expansion and enlargement during berry development [42-44]. To provide additional insights into the transcriptional regulation of cell wall metabolism during grapevine development, co-expression analysis using a grapevine bHLH TF, grapevine CEB1, which is known to regulate grape berry development [42] was performed. In this example, the respective unique code for grapevine CEB1 (VIT_01s0244g00010) was input into the ‘CoexQuery’ field and selected ‘all’ and ‘HRR’ for the preferred predefined datasets and co-expression measure options, respectively (Figure 1B) or by using the keyword query ‘CEB1’ (Figure 1C) and choosing ‘all’ under the ‘CoexQuery’ column in the ‘keyword query’ results page. A total of 266 genes were indicated to be co-expressed with grapevine CEB1, with average HRR and PCC values of 167 and 0.73 respectively. Among others, genes encoding enzymes involved in auxin signalling (SAUR9, VIT_04s0023g03230; ARF2_2, VIT_01s0244g00150; TPR1, VIT_04s0008g06400), cell wall metabolism (EXPA11, VIT_18s0001g01130) and various classes of TF (bHLH, ERF, MYB) were highly co-expressed with grapevine *VvCEB1* (Additional file 3: Table S1). In agreement with the co-expression results, experimental evidence has shown that overexpression of VvCEB1 in grapevine embryos is able to stimulate cell expansion via control of Auxin/IAA TFs, SAUR and cell wall modification genes [42]. Interestingly, among the transcripts tested, EXPA11 (VIT_18s0001g01130, XM_002285855.1 in their study) was the most up-regulated (> 1000 fold) in VvCEB1-overexpressing grape embryos compared to control [42]. In the co-expressed genes results, EXPA11 (VIT_18s0001g01130) was highly co-expressed (top 6) with grapevine *VvCEB1* in ‘all’ conditions while the HRR (top 1) were improved in berry-related datasets (Figure 3C). As an alternative, selecting other predefined conditions to understand the molecular mechanisms of query genes in a specific context could also be carried out. Nevertheless, using ‘all’ datasets is sufficient for most applications. Below the list of co-expressed genes, GO enrichment analysis of the whole co-expressed gene lists (HRR <350, *P* < 0.01) revealed that terms such as GO:0006355, regulation of transcription DNA-dependent (5.06e-05); GO:0009699, phenylpropanoid biosynthetic process (3.86e-06); GO:0009813, flavonoid biosynthetic process (8.57e-04) and GO:0009745, sucrose mediated signalling (3.95e-04) were highly enriched (Figure 3D; Additional file 3: Table S2). This data suggests the potential involvement of additional TFs, phenylpropanoid (shikimate) pathway genes and sucrose metabolism in regulating the cell expansion during berry development. Additionally, the highly interactive expression specificity chart showed that the grapevine *VvCEB1* gene was expressed specifically in berry-related tissues with highest specificity during veraison onwards (Figure 3E; Additional file 3: Table S3). Taken together, the co-expression results largely confirm results from previous studies and strengthen the putative role of *VvCEB1* in controlling berry growth while providing additional clues into the complex molecular mechanisms of VvCEB1 [i.e. potential targets (direct/indirect), expression specificity and enriched pathways].

### Example application II: photosynthesis and phenylpropanoid metabolism

**Figure 5:**
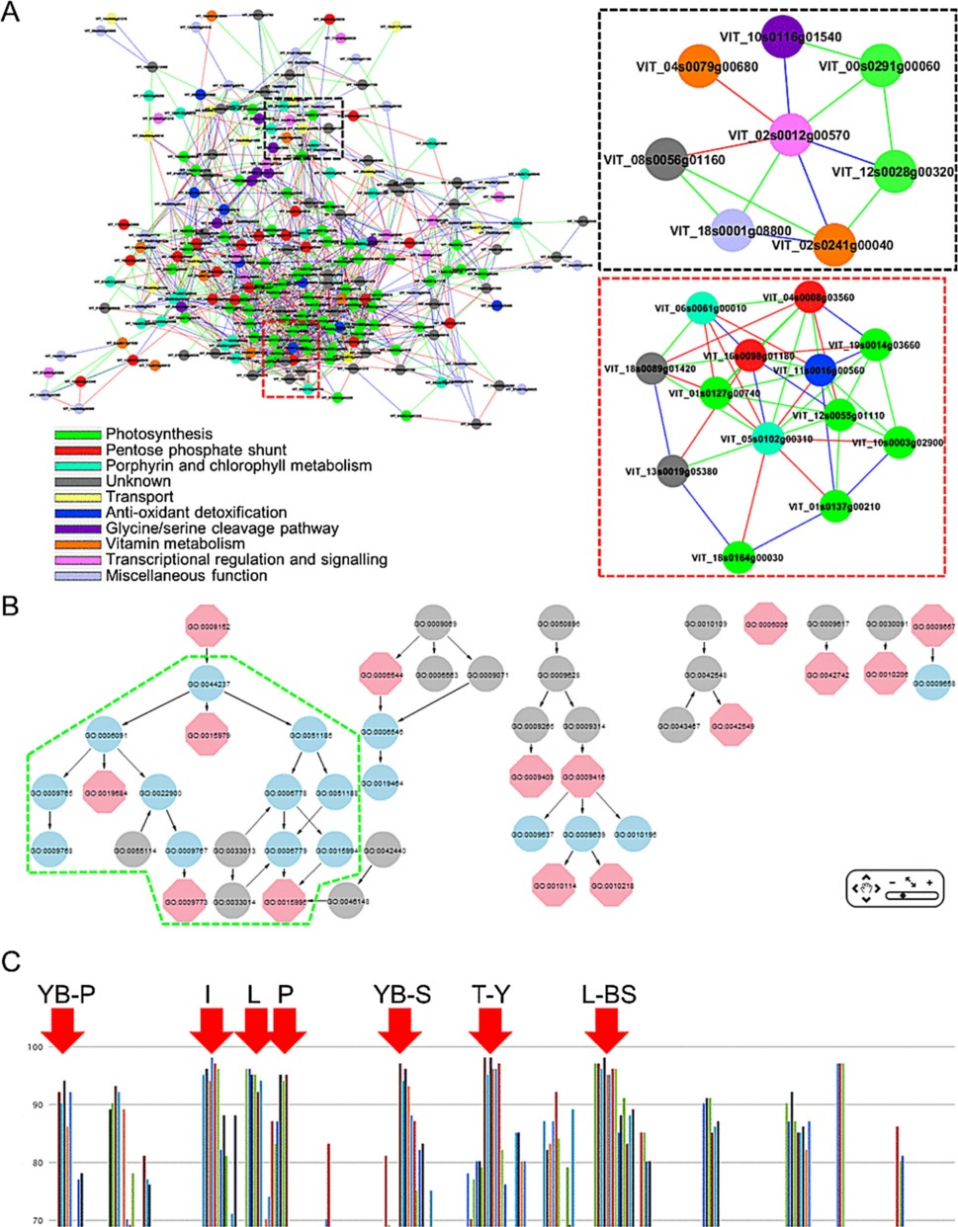
**Predicted module involved in photosynthesis (Module All_MCL_6). (A)** Output for module ‘All_MCL_6’, which contains 240 nodes and 1419 edges, with genes encoding proteins predominantly involved in photosynthesis (green), pentose-phosphate pathway (red), porphyrin and chlorophyll metabolism (cyan), transport (yellow), anti-oxidant detoxification (blue) and vitamin metabolism (orange) while others were involved in glycine/serine cleavage pathway (purple), transcriptional regulation and signalling (pink) and miscellaneous functions (light blue). Grey nodes represent genes whose function remains unknown or uncharacterised based on homology searches. Sub-network of putative APRR2 and GUN4 extracted from module ‘All_MCL_6’ within a neighbourhood distance of 1 (i.e. nodes connected directly to the query gene) are highlighted in black and red boxes, respectively. **(B)** Network representation of truly significant and highly enriched GO:BP terms related to photosynthesis, electron transport and chlorophyll metabolism are highlighted in green boxes. Other enriched GO terms were removed from presentation for clarity. **(C)** Expression specificity of module ‘All_MCL_6’ showing experiments in which at least 70% of the probesets demonstrated specific expression (Std2Gx > 1.0). YB-P, young berries (pericarp); I, inflorescence; L, leaves; P, petals; YB-S, young berries (skin); T, tendrils (young); L-BS, leaves-biotic stress.

When *a priori* knowledge of a target gene is not known, searches using terms of interest enriched within predicted modules can be conducted using VTCdb. Users can query broad GO terms (ID/description) such as GO:0019684 or ‘photosynthesis, light reaction’ (Figure 1D) and select the preferred condition and graph clustering approach in the ‘enrich module results’ page (Figure 2D). In this example, when a condition-independent (all) and MCL approach is selected, modules (ALL_MCL) 6, 21, 233 and 133 were enriched with GO:0019684 (photosynthesis, light reaction) with module All_MCL_6 having the highest adjusted *P*-values of 4.28E-42. Upon browsing All_MCL_6, it could be seen that the module showed 240 nodes, of which 68 had predicted roles related to photosynthesis (~30%), while some were involved in antioxidant detoxification and were predicted components of the chloroplast ascorbate-glutathione cycle [45] (Figure 5A). Such genes included those encoding chloroplastic ascorbate peroxidase (VIT_18s0001g06370 and VIT_04s0008g05490), peroxiredoxin (VIT_11s0016g00560), thioredoxin (VIT_18s0001g00820, VIT_18s0001g10510 and VIT_18s0001g15310) and glutathione S-transferase (VIT_06s0004g03690). Furthermore, genes encoding proteins involved in electron transport, tetrapyrrole metabolism, the pentose phosphate pathway, glycine/serine cleavage system and vitamin metabolism were clustered with photosynthesis-related genes (Figure 5A, Additional file 3: Table S4). As anticipated, truly significant and highly enriched ontological terms for biological processes (GO: BP) such as GO:0015979, photosynthesis (6.87E-77); GO:0015995, chlorophyll biosynthetic process (5.92E-08); GO:0009773, photosynthetic electron transport (8.30E-11) and GO:0006544, glycine metabolic process (1.48E-09) were highly enriched within this module (Figures 4B and 5B, Additional file 3: Table S5). This observation is consistent with the many co-expression studies previously conducted in *Arabidopsis*, where genes involved in photosynthesis and related processes were found to form well-defined co-expression modules [46]. This is likely because photosynthesis requires the coordinated assembly of proteins into large super-complexes with numerous protein-protein interactions, and therefore their corresponding genes are expected to be highly co-expressed [47]. Interestingly, the sub-network of a putative grapevine GUN4 (VIT_05s0102g00310) was connected to genes encoding proteins involved in photosynthesis (i.e. LHCB6, VIT_12s0055g01110; LHC2 type 1, VIT_10s0003g02900) and tetrapyrrole metabolism (CHLD, VIT_06s0061g00010), corroborating the role of *GUN4* in regulating photosynthesis and chlorophyll development at the post-translational level [48] (Figure 5A). Additionally, a node annotated as a putative APRR2 (VIT_02s0012g00570) within this module was connected to several photosynthesis-related genes (LHB1B1, VIT_12s0028g00320 and PSBW, VIT_14s0081g00060) and vitamin E metabolism (VTE5, VIT_13s0074g00040 and PSY, VIT_04s0079g00680) which was in agreement with the functional role of APRR2 in co-regulating genes primarily involved in photosynthesis and chloroplast functions, resulting in increased plastid size, chlorophyll content and pigmentation in plants [49] (Figure 5A). While the majority of nodes within this module were successfully annotated, there are several nodes in which no clear annotation has been ascribed by homology searches and which can nonetheless be hypothesized to be involved in photosynthesis-related functions given their tight co-expression relationships (Figure 5A) [50]. The expression specificity of All_MCL_6 also indicated that genes within the module were specifically expressed in leaves, seedlings, tendril, inflorescence and young berries, while expression levels were very low in post-veraison berries, seeds and callus samples (Figures 4C and 5C, Additional file 3: Table S6). This is not unexpected, considering their functional roles in photosynthesis, and their association with the chloroplast and photosynthetic tissues. At the cluster level (meta-network), module All_MCL_6 was significantly connected (*P* < 0.01) to many modules including (All_MCL) 238, 389, 156, 416, 233 and 23 (Additional file 3: Table S7). GO BP functional analysis of the latter modules found that they were highly enriched with translation, redox metabolism, photosynthesis and tetrapyrrole metabolism terms. Similar to the enrichment of biological processes within modules, connected modules may participate in related processes given they share a significant proportion of co-expressed gene pairs and may provide additional information on the organization and coordination of module within the meta-network of co-expressed genes. From these data, it seems likely that genes constituting module All_MCL_6 are involved in the maintenance of redox regulation and homeostasis in the chloroplast thylakoids during photosynthesis and related processes.

**Figure 6:**
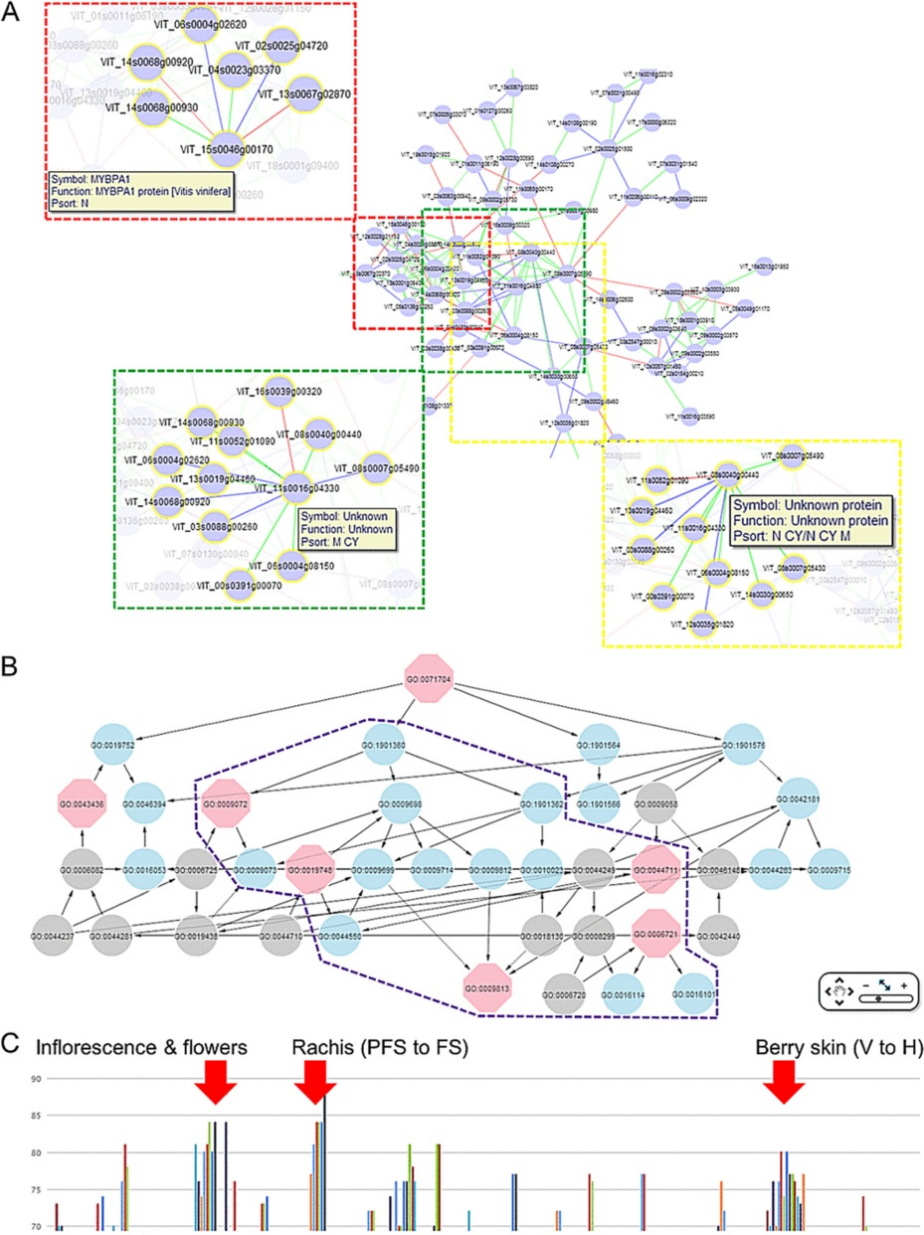
**Predicted module involved in flavonoid, amino acid and related metabolism (module All _HCCA_181). (A)** Output for module ‘All _HCCA_181’, which contains 74 nodes and 152 edges, includes genes predominantly associated with general flavonoid, aromatic amino acid metabolic pathways and transport. Sub-network of putative grapevine MYBPA1 and two unknown genes extracted from module ‘All _HCCA_181’ within a neighbourhood distance of 1 are highlighted in red, green and yellow boxes, respectively. **(B)** Network representation of truly significant and highly enriched GO:BP terms related to flavonoid, aromatic amino acid and terpenoid metabolism are highlighted in purple boxes. Other enriched GO terms were removed from presentation for clarity. **(C)** Expression specificity of module ‘All _HCCA_181’ showing experiments in which the probesets (70% or greater) demonstrated specific expression (Std2Gx > 1.0). PFS, post fruitset; FS, fruitset; V, veraison, H, harvest.

To search for interesting modules involved in phenylpropanoid metabolism, a query using broad keyword terms such as “GO:0009698” or “phenylpropanoid metabolic process” and selecting the preferred condition (all datasets and HCCA clustering), returned a list of modules in which the query term was enriched (Additional file 3: Figure S1). Further inspection showed that members within respective modules (HCCA) were involved in various specialised pathways downstream of the main phenylpropanoid pathway, such as stilbenoid biosynthesis (module All_HCCA_60), flavonoid biosynthesis (module All_HCCA_181), lignin/lignan metabolism (modules All_HCCA_65 and All_HCCA_139), and the hypersensitive response (modules All_HCCA_131 and All_HCCA_186). Natural products derived from the phenylpropanoid pathway play various fundamental roles in plants, including protection against abiotic stress, plant-pathogen/herbivore interaction and plant development. These secondary metabolites can encompass various classes of anthocyanins, flavonols, proanthocyanidins, lignins, terpenes and stilbenes. In this example we describe, in greater detail, how useful information could be obtained from module All_HCCA_181 (Figure 6). This module contained 74 nodes and 152 edges, represented predominantly by structural genes encoding enzymes of the flavonoid biosynthetic pathway (flavonone 3-hydroxylase, VIT_04s0023g03370; dihydroflavonol 4-reductase, VIT_18s0001g12800; leucoanthocyanidin dioxygenase, VIT_02s0025g04720), shikimate pathway (shikimate dehydrogenase, VIT_14s0030g00650; chorismate mutase, VIT_14s0108g01330; 3-deoxy-D-arabino-heptulosonate 7-phosphate synthase, VIT_00s0391g00070), TFs (MYBPA1, VIT_15s0046g00170) and transferase/transport proteins (Transparent testa 12, VIT_12s0028g01150) (Figure 6A, Additional file 3: Table S8). GO enrichment showed that GO:BP terms such as flavonoid biosynthetic process (GO:0009813; SCS < 2.14e-10) and aromatic amino acid family metabolic process (GO:0009072; SCS < 6.54e-06) were significantly enriched as anticipated (Figure 6B; Additional file 3: Table S9). Furthermore, the sub-network surrounding the grapevine TF MYBPA1 in module All_HCCA_181 showed that the TF was linked to structural genes of the flavonoid metabolic pathway, corroborating previous gene expression and promoter studies [51,52] (Figure 6A). The majority of genes from module All_HCCA_181 (>70%) were specifically expressed in berry skins (during véraison and ripening), flowers, buds, inflorescence, buds and rachis (FS and PFS) coinciding with the tissues and developmental programming of flavonoid accumulation [53] (Figure 6C, Additional file 3: Table S10). Additionally, module All_HCCA_181 was significantly connected (*P* < 0.01) to modules (All_HCCA) 13, 90, 60, 242 (Additional file 3: Table S11). Functional analysis (GO:BP) of the latter modules suggested that they were highly enriched with ontologies involved in fatty acid (lipids, steroids, wax), hormone, anthocyanin and stilbenoid metabolic processes. Also, there was enrichment for genes involved in stress responses, which taken together suggests a strong coordination between modules enriched within the large family of phenylpropanoid biosynthetic process. Many of the genes implicated in the flavonoid pathway, including regulatory genes, have been identified here and in previous work. However, several nodes annotated as proteins of unknown function (such as VIT_11s0016g04330 and VIT_08s0040g00440), may also qualify as candidates for biosynthetic or regulatory gene products of this specialised pathway, based on their dense connectivity with genes of the flavonoid and aromatic amino acid metabolic pathway and shared expression patterns. Such genes could be potential candidates for future characterisation of phenylpropanoid metabolic and regulatory networks (Figure 6A and C).

The regulation of genes associated with photosynthesis and flavonoid metabolism displayed conserved co-expression network structures at the gene and module level across nine different plant species [21,28]. Both MCL and HCCA were able to partition the grapevine co-expression network efficiently and into biologically relevant modules in which genes involved in shared biological processes were successfully recovered. Thus, the co-expression analysis (both condition-independent and -dependent) performed here largely confirms previous work while revealing new putative roles for uncharacterised grapevine genes, and demonstrates the utility of the grapevine co-expression network generated in this study.

### Comparison to similar co-expression studies and future developments

Currently, two other broad plant co-expression databases include grapevine microarray data [23,28]. Compared with these, VTCdb has a species-specific focus on grapevine and offers additional advantages including (1) greater transcriptome coverage for GCA, encompassing over 29,000 genes (>95% of the predicted genome, according to the12X v1 grape gene annotation), (2) flexibility to perform GCA based on either the 29 K Nimblegen array or the extensively utilised 16 K Affymetrix Genechip, (3) the option to choose between ‘condition-independent’ and ‘condition-dependent’ GCA, (4) the option to explore grapevine functional modules inferred from various graph clustering approaches and (5) the provision of web-based tools to enhance the functional interpretation from GCA (i.e. functional enrichment analysis, expression patterns across a wide range of experimental conditions/treatments and network visualisation). We note that despite having thorough transcriptome coverage, this study can only provide a glimpse into ‘condition-specific’ GCA in grapevine. Arrays of experimental conditions encompassing berry tissues and berry developmental series as well as limited stress conditions and management treatments were sufficiently represented in the public domain. A comprehensive catalogue for datasets encompassing additional stress, hormone and tissue datasets is still needed to fine-tune and facilitate the discovery of novel co-expression relationships based on condition-specific circumstances. To this end, biannual updates of the database will be conducted when new microarray experiments are published or sufficient arrays from other platforms becomes available for GCA. Nevertheless, users of VTCdb are able to perform GCA using datasets from the 16 K Affymetrix Genechip, which encompass a greater variety of experimental conditions (e.g. drought, salinity, heat and pathogen attack) than the Nimblegen array, albeit at the cost of transcriptome coverage. We have demonstrated that the co-expression relationships obtained using grapevine berry development, photosynthesis and flavonoid pathway-related genes were robust and could be used to identify novel transcriptional regulatory mechanisms, supported by combined network and functional analysis in plants [49,51,54]. These are examples of how VTCdb can be utilised to infer gene function. The predicted modules using graph clustering were of high biological relevance and may offer new biological insights into many uncharacterised genes within these modules. Due to the large proportion of uncharacterised genes within the grapevine genome, functional annotation on the basis of gene co-expression analysis and expression patterns will provide an additional tool toward gene discovery. Therefore, VTCdb offers a one-stop online platform for GCA for the grapevine research community.

## Conclusions

Gene co-expression analysis of the grapevine transcriptome and the creation of an online tool to interrogate this data, provide a vital step towards uncovering additional relationships using publicly available grapevine microarray data. This meta-analysis approach has facilitated the comprehensive annotation of functions to unknown genes and the discovery of functional modules in grapevines. With the rising trend of transcriptional analyses using RNA-sequencing in grapevine [55-57] and on-going improvement of the methods required to process these data for GCA [58,59], the prospect for GCA using grapevine RNA-sequencing data will become feasible in the future. Nevertheless, for the purposes of reverse-engineering gene co-expression networks, microarrays are currently better suited in this goal [58]. We envisage the utility and potential of VTCdb (http://vtcdb.adelaide.edu.au/home.aspx) to provide further valuable information in hypothesis-driven studies and to aid grapevine researchers in their prioritisation of gene candidates for further study towards the understanding of biological processes related to many aspects of grapevine development and metabolism.

## Availability and requirements

All results discussed within this study and additional tools to query pre-constructed networks, perform additional gene co-expression, expression meta-analysis and annotation searches are available freely at VTCdb (http://vtcdb.adelaide.edu.au/home.aspx). VTCdb supports all major web-browsers, preferably Google Chrome or Mozilla Firefox for visualization and performance purposes.

## Abbreviations

VTCdb: Vitis Transcriptomics and co-expression database; GCA: Gene co-expression analysis; GCN: Gene co-expression network; TF: Transcription factor; GO: Gene ontology; PCC: Pearson’s correlation coefficient; SCC: Spearman’s correlation coefficient; HRR: Highest reciprocal rank; MR: Mutual rank; SCS: Set count sizes; HCCA: Heuristic cluster chiselling algorithm; MCL: Markov clustering.

## Competing interests

The authors declare that they have no competing interests.

## Authors’ contributions

DCJW conceived the study, compiled and analysed the microarray data, performed co-expression data analysis, constructed the database platform and drafted the manuscript. CMF, CS, DPD participated in co-expression data analysis, design and coordination of the study and assisted in drafting the manuscript. All authors have read and approved the final manuscript.

## Supplementary Material

Additional file 1 **Microarray datasets and associated meta-data used in the construction of the grape co-expression network.** A brief description pertaining to the unique accession, title, and number of assays, reference and conditions of microarray datasets from 16 K Affymetrix Genechip and 29 K Nimblegen whole-genome array are listed in Tables S1 and S2, respectively. Figure S1 contains screenshots of the fully expanded VTCdb web interface containing various forms to perform gene co-expression analysis. Click here for file

Additional file 2 **A detailed description of the microarray datasets and associated meta-data used in the construction of the grape co-expression network from 16 K Affymetrix Genechip and 29 K Nimblegen whole-genome array are listed in sheets according to the respective PlexDB ID (for 16 K Affymetrix Genechip array) and GEO/Arrayexpress ID (for 29 K Nimblegen whole-genome array).** Experiments that were considered potential outliers were highlighted in red. Click here for file

Additional file 3 **Excel file containing twelve worksheets with a list of co-expressed genes (HRR ≤ 350, *P*-value < 0.01) for grapevine CEB1 (Table S1), a list of all GO terms enriched associated with CEB1 co-expressed genes (Table S2), a table containing the expression specificity of CEB1 (Table S3), a list of genes and associated information from module All_MCL_6 (Table S4), a list of all GO terms enriched in module All_MCL_6 (Table S5), a table containing the expression specificity of module All_MCL_6 (Table S6), a list containing the significantly connected clusters to module All_MCL_6 (Table S7), screenshots of the utility and output of keyword query using ‘GO:0009698’ as input (Figure S1), a list of genes and associated information from module All_HCCA_181 (Table S8), and a list of all GO terms enriched in module All_HCCA_181 (Table S9), a table containing the expression specificity of module All_HCCA_181 (Table S10), a list containing the significantly connected clusters to module All_HCCA_181 (Table S11).** Click here for file
