# Supplementary material for: VTCdb: a gene co-expression database for the crop species Vitis vinifera (grapevine)
Source: BMC Genomics. 2013 Dec 16;14:882. doi: 10.1186/1471-2164-14-882 (PMC3904201; doi:10.1186/1471-2164-14-882)
Supplement: Additional file 1 — Microarray datasets and associated meta-data used in the construction of the grape co-expression network. A brief description pertaining to the unique accession, title, and number of assays, reference and conditions of microarray datasets from 16 K Affymetrix Genechip and 29 K Nimblegen whole-genome array are listed in Tables S1 and S2, respectively. Figure S1 contains screenshots of the fully expanded VTCdb web interface containing various forms to perform gene co-expression analysis. [file 1471-2164-14-882-S1.docx]

Table 1: 451 microarray (16K Affymetrix Genechip) datasets across 20 experiments

| Exp ID | Title | Assays | Ref | Conditions | Summary |
| --- | --- | --- | --- | --- | --- |
| VV40 | Transcriptomic analysis during heat stress and after the following recovery in grapevine (Vitis vinifera L.) leaves | 12 | [[1](#_ENREF_1)] | temperature | •45 C •25 C •45-25 C •25-25 C |
| VV31 | Expression data from 35S::VvCBF4-overexpressing grapevines | 8 | [[2](#_ENREF_2)] | genetic line | •VvCBF4 overexpressor •control |
| VV29 | Expression data from micropropagated Vitis vinifera when transferred to ex vitro conditions | 4 | [[3](#_ENREF_3)] | time | •0 hrs •48 hrs |
| VV28 | Gene expression associated with compatible viral diseases in berry | 14 | [[4](#_ENREF_4)] | pathogen infection | •GLRaV-3 virus-infected •Virus-free |
|  |  |  |  | developmental stage name | •Veraison •Ripening |
| VV17 | Grape skin transcriptome in the berries cultured in vitro treated with exogenous abscisic acid. | 8 | - | treated or untreated  time | •ABA treated •Non-treated (control) |
|  |  |  |  |  | •3 days •10 days |
| VV16 | Grape skin transcriptome in the berries grown on the vine treated with exogenous abscisic acid | 12 | - | treated or untreated | •ABA treated •untreated (control) |
|  |  |  |  | developmental stage name | •14 days after veraison •28 days after veraison |
| VV15 | Expression data in individual grape berries during ripening initation | 32 | [[5](#_ENREF_5)] | developmental stage name | •green hard •green soft •pink soft •red soft |
|  |  |  |  | berry position | •distal •proximal |
|  |  |  |  | plant | •1 •2 |
| VV14 | Gene expression in grapevine in response to Bois noir infection | 10 | [[6](#_ENREF_6)] | genotype | •Chardonnay •Incrocio Manzoni |
|  |  |  |  | disease type | •Bois Noir infected •Healthy |
| VV12 | Powdery Mildew-Induced Transcriptome in a Susceptible Grapevine ‘Cabernet Sauvignon’ | 36 | [[7](#_ENREF_7)] | treated or untreated | •PM-inoculated •mock-inoculated |
|  |  |  |  | time | •0 hai •4 hai •8 hai •12 hai •24 hai •48 hai |
| VV11 | Pinot Noir berry transcriptome during ripening. | 27 | [[8](#_ENREF_8)] | year | •2003 •2005 •2006 |
|  |  |  |  | developmental stage | •33E-L, •34E-L, •36E-L, |
| VV9 | High temperature effect on Cabernet Sauvignon berries | 12 | - | developmental stage | •2 weeks •4 weeks •6 weeks |
|  |  |  |  | temperature | •High temperature •Control |
| VV7 | Gene expression associated with compatible viral diseases in grapevine cultivars | 6 | [[4](#_ENREF_4)] | biotic stress | •virus infection •control |
| VV5 | Chardonnay and Cabernet-Sauvignon's berry development | 84 | [[9](#_ENREF_9)] | developmental stage | •31 •32 •33 •34 •35 •36 •38 |
|  |  |  |  | Treated or untreated | •WW •WD |
|  |  |  |  | cultivar | •Cab •Chard |
| VV3 | Grape berry tissues differentiation | 18 | [[10](#_ENREF_10)] | tissue type | •Pulp •Skin •Seed |
|  |  |  |  | Treated or untreated | •Well watered •Water deficit |
| VV2 | Long-term Salt & Water Stress in Grapes | 39 | [[11](#_ENREF_11)] | time | •Day 0 •Day 4 •Day 8 •Day 12 •Day 16 |
|  |  |  |  | stress | •Control •Water-Deficit •Salinity |
| VV1 | short term abiotic stress Cabernet Sauvignon | 48 | - | time | •0 h •1 h •4 h •8 h •24 h |
|  |  |  |  | treatment | •unstressed •salt •PEG •cold (5 C) |
| GSE44213 | Transcriptional responses to water deficit and Xylella fastidiosa (Pierce's disease) in Vitis vinifera | 45 | [[12](#_ENREF_12)] | biotic stress | •Bacteria infection •control |
|  |  |  |  | Treated or untreated | •Well watered •Water deficit |
| E-MEXP-1950 | Transcription profiling by array of grape cultivar Carignan RPM variants | 12 | [[13](#_ENREF_13)] | genotype | •RRM •Carignan |
|  |  |  |  | tissue | •Inflorescence |
| E-MEXP-1524 | Transcription profiling of grapevine cell culture treated with methyly jasmonate, salicylic acids and ethanol controls | 12 | [[14](#_ENREF_14)] | tissue | •cell culture |
|  |  |  |  | Treated or untreated | •MeJA •SA •Ethanol •control |
| E-MEXP-3045 | Sulphur dioxide evokes large scale reprogramming of the grape berry transcriptome | 12 | [[15](#_ENREF_15)] | Treated or untreated | •SO2 •SA •MeJA •SA+MeJA•control |
|  |  |  |  | tissue | •post-harvest berries |

Table 2: 418 microarray (29K Nimblegen whole-genome) datasets; 475 experiments datasets across 8 experiments

| Accession | Title | Assay | Ref | Factor | Summary |
| --- | --- | --- | --- | --- | --- |
| E-MEXP-3803 | Transcription profiling by array of Vitis vinifera Cabernet Sauvignon auto-grafts to investigate genes differentially expressed in the rootstock and callus | 10 | [[16](#_ENREF_16)] | Tissue | •Rootstocks •Callus |
|  |  |  |  | Time after grafting | •3d •28d |
| GSE32343 | Grapevine response to Planococcus ficus feeding | 8 | - | treated or untreated | •PF-inoculated •Control |
|  |  |  |  | Time | •6h •96h |
| GSE34634 | Sauvignon blanc berry developmental | 7 | - | Time | •Green •Veraison •Harvest |
| GSE36128 | The grapevine expression atlas reveals a deep transcriptome shift driving the entire plant into a maturation program | 162 | [[17](#_ENREF_17)] | Tissue | •Various tissues (Stamen,BerryPericarp-FS,BerryPericarp-PFS,BerryPericarp-V,BerryPericarp-MR,BerryPericarp-R,Bud-S,Bud-B,Bud-AB,Bud-L,Bud-W,BerryFlesh-PFS,BerryFlesh-V,BerryFlesh-MR,BerryFlesh-R,BerryFlesh-PHWI,BerryFlesh-PHWII,BerryFlesh-PHWIII,Inflorescence-Y,Inflorescence-WD,Flower-FB,Flower-F,Root,Leaf-Y,Leaf-FS,Leaf-S,Carpel,Petal,BerryPericarp-PHWI,BerryPericarp-PHWII,BerryPericarp-PHWIII,Pollen,Rachis-FS,Rachis-PFS,Rachis-V,Rachis-MR,Rachis-R,Seed-V,Seed-MR,Seed-FS,Seed-PFS,Seedling,BerrySkin-PFS,BerrySkin-V,BerrySkin-MR,BerrySkin-R,BerrySkin-PHWI,BerrySkin-PHWII,BerrySkin-PHWIII,Stem-G,Stem-W,Tendril-Y,Tendril-WD,Tendril-FS) |
| GSE36234 | Increasing the source/sink ratio in Vitis vinifera (cv Sangiovese) induces extensive transcriptome reprogramming and modifies berry ripening | 18 | [[18](#_ENREF_18)] | treated or untreated | •Cluster-thinned •Control |
|  |  |  |  | Time | •Beginning-Veraison •End-Veraison •Harvest |
| GSE36632 | Co-evolution between Grapevine rupestris stem pitting-associated virus and Vitis vinifera L. induces a decrease in defence responses and physiological performance associated with an increase in photosynthesis-related gene transcription | 18 | [[19](#_ENREF_19)] | Tissue | •petioles •leaves •berries-Veraison |
|  |  |  |  | treated or untreated | •GRSPaV-infected •Control |
| GSE40487 | Selective defoliation affects plant growth, fruit transcriptional ripening program and flavonoid metabolism in grapevine. | 24 | [[20](#_ENREF_20)] | treated or untreated | •Defoliation •Control |
|  |  |  |  | Time | •Beginning-Veraison •End-Veraison •Harvest |
| GSE49569 | Plasticity of the ripening process among berry classes | 63 | - | Time | •prevéraison •midvéraison •Harvest |
|  |  |  |  | Tissue | •Skin •Seed •Pulp |
| GSE41633 | The plasticity of the grapevine berry transcriptome | 171 | [[21](#_ENREF_21)] | Time | •Veraison •Mid-ripening •Harvest |
|  |  |  |  | Microclimate/farming | •altitude •soil-type •training system •rows facing direction •planting layout •vineyard age •rootstock type |
|  |  |  |  | Year | •2006 •2007 •2008 |

**References**

1. Liu G-T, Wang J-F, Cramer G, Dai Z-W, Duan W, Xu H-G, Wu B-H, Fan P-G, Wang L-J, Li S-H: **Transcriptomic analysis of grape (Vitis vinifera L.) leaves during and after recovery from heat stress**. *BMC Plant Biology* 2012, **12**(1):174.

2. Tillett RL, Wheatley MD, Tattersall EAR, Schlauch KA, Cramer GR, Cushman JC: **The Vitis vinifera C-repeat binding protein 4 (VvCBF4) transcriptional factor enhances freezing tolerance in wine grape**. *Plant Biotechnology Journal* 2012, **10**(1):105-124.

3. Carvalho LC, Vilela BJ, Mullineaux PM, Amâncio S: **Comparative Transcriptomic Profiling of Vitis vinifera Under High Light Using a Custom-Made Array and the Affymetrix GeneChip**. *Molecular Plant* 2011, **4**(6):1038-1051.

4. Vega A, Gutiérrez R, Peña-Neira A, Cramer G, Arce-Johnson P: **Compatible GLRaV-3 viral infections affect berry ripening decreasing sugar accumulation and anthocyanin biosynthesis in Vitis vinifera**. *Plant Mol Biol* 2011, **77**(3):261-274.

5. Lund S, Peng F, Nayar T, Reid K, Schlosser J: **Gene expression analyses in individual grape (Vitis vinifera L.) berries during ripening initiation reveal that pigmentation intensity is a valid indicator of developmental staging within the cluster**. *Plant Mol Biol* 2008, **68**(3):301-315.

6. Albertazzi G, Milc J, Caffagni A, Francia E, Roncaglia E, Ferrari F, Tagliafico E, Stefani E, Pecchioni N: **Gene expression in grapevine cultivars in response to Bois Noir phytoplasma infection**. *Plant Science* 2009, **176**(6):792-804.

7. Fung RWM, Gonzalo M, Fekete C, Kovacs LG, He Y, Marsh E, McIntyre LM, Schachtman DP, Qiu W: **Powdery Mildew Induces Defense-Oriented Reprogramming of the Transcriptome in a Susceptible But Not in a Resistant Grapevine**. *Plant Physiology* 2008, **146**(1):236-249.

8. Pilati S, Perazzolli M, Malossini A, Cestaro A, Dematte L, Fontana P, Dal Ri A, Viola R, Velasco R, Moser C: **Genome-wide transcriptional analysis of grapevine berry ripening reveals a set of genes similarly modulated during three seasons and the occurrence of an oxidative burst at veraison**. *BMC Genomics* 2007, **8**(1):428.

9. Deluc L, Grimplet J, Wheatley M, Tillett R, Quilici D, Osborne C, Schooley D, Schlauch K, Cushman J, Cramer G: **Transcriptomic and metabolite analyses of Cabernet Sauvignon grape berry development**. *BMC Genomics* 2007, **8**(1):429.

10. Grimplet J, Deluc LG, Tillett RL, Wheatley MD, Schlauch KA, Cramer GR, Cushman JC: **Tissue-specific mRNA expression profiling in grape berry tissues**. *BMC Genomics* 2007, **8**:187.

11. Cramer G, Ergul A, Grimplet J, Tillett R, Tattersall E, Bohlman M, Vincent D, Sonderegger J, Evans J, Osborne C: **Water and salinity stress in grapevines: early and late changes in transcript and metabolite profiles**. *Funct Integr Genomics* 2007, **7**(2):111 - 134.

12. Choi H-K, Iandolino A, da Silva FG, Cook DR: **Water Deficit Modulates the Response of Vitis vinifera to the Pierce's Disease Pathogen Xylella fastidiosa**. *Molecular Plant-Microbe Interactions* 2013, **26**(6):643-657.

13. Fernandez L, Torregrosa L, Segura V, Bouquet A, Martinez-Zapater JM: **Transposon-induced gene activation as a mechanism generating cluster shape somatic variation in grapevine**. *The Plant Journal* 2010, **61**(4):545-557.

14. Onofrio CD, Cox A, Davies C, Boss PK: **Induction of secondary metabolism in grape cell cultures by jasmonates**. *Functional Plant Biology* 2009, **36**(4):323-338.

15. Giraud E, Ivanova A, Gordon CS, Whelan J, Considine MJ: **Sulphur dioxide evokes a large scale reprogramming of the grape berry transcriptome associated with oxidative signalling and biotic defence responses**. *Plant, Cell & Environment* 2012, **35**(2):405-417.

16. Cookson SJ, Clemente Moreno MJ, Hevin C, Nyamba Mendome LZ, Delrot S, Trossat-Magnin C, Ollat N: **Graft union formation in grapevine induces transcriptional changes related to cell wall modification, wounding, hormone signalling, and secondary metabolism**. *Journal of Experimental Botany* 2013.

17. Fasoli M, Dal Santo S, Zenoni S, Tornielli GB, Farina L, Zamboni A, Porceddu A, Venturini L, Bicego M, Murino V *et al*: **The Grapevine Expression Atlas Reveals a Deep Transcriptome Shift Driving the Entire Plant into a Maturation Program**. *The Plant Cell Online* 2012, **24**(9):3489-3505.

18. Pastore C, Zenoni S, Tornielli GB, Allegro G, Dal Santo S, Valentini G, Intrieri C, Pezzotti M, Filippetti I: **Increasing the source/sink ratio in Vitis vinifera (cv Sangiovese) induces extensive transcriptome reprogramming and modifies berry ripening**. *BMC Genomics* 2011, **12**(1):631.

19. Gambino G, Cuozzo D, Fasoli M, Pagliarani C, Vitali M, Boccacci P, Pezzotti M, Mannini F: **Co-evolution between Grapevine rupestris stem pitting-associated virus and Vitis vinifera L. leads to decreased defence responses and increased transcription of genes related to photosynthesis**. *Journal of Experimental Botany* 2012, **63**(16):5919-5933.

20. Pastore C, Zenoni S, Fasoli M, Pezzotti M, Tornielli GB, Filippetti I: **Selective defoliation affects plant growth, fruit transcriptional ripening program and flavonoid metabolism in grapevine**. *BMC Plant Biol* 2013, **13**:30.

21. Dal Santo S, Tornielli G, Zenoni S, Fasoli M, Farina L, Anesi A, Guzzo F, Delledonne M, Pezzotti M: **The plasticity of the grapevine berry transcriptome**. *Genome Biology* 2013, **14**(6):r54.
